# PTEN expression is a prognostic marker for patients with non-small cell lung cancer: a systematic review and meta-analysis of the literature

**DOI:** 10.18632/oncotarget.11068

**Published:** 2016-08-05

**Authors:** Jian Xiao, Cheng-Ping Hu, Bi-Xiu He, Xi Chen, Xiao-Xiao Lu, Ming-Xuan Xie, Wei Li, Shu-Ya He, Shao-Jin You, Qiong Chen

**Affiliations:** ^1^ Department of Geriatrics, Respiratory Medicine, Xiangya Hospital of Central South University, Changsha 410008, China; ^2^ Department of Respiratory Medicine, Xiangya Hospital of Central South University, Changsha 410008, China; ^3^ Department of Biochemistry and Biology, University of South China, Hengyang 421001, China; ^4^ Laboratory of Cancer Experimental Therapy, Atlanta Research and Educational Foundation (151F), Atlanta VA Medical Center, Decatur, GA 30033, USA

**Keywords:** non-small cell lung cancer, NSCLC, PTEN, disease free survival, DFS

## Abstract

Phosphatase and tensin homolog deleted on chromosome 10 (PTEN) is a known tumor suppressor in non-small cell lung cancer (NSCLC). By performing a systematic review and meta-analysis of the literature, we determined the prognostic value of decreased PTEN expression in patients with NSCLC. We comprehensively and systematically searched through multiple online databases up to May 22, 2016 for NSCLC studies reporting on PTEN expression and patient survival outcome. Several criteria, including the Newcastle-Ottawa Quality Assessment Scale (NOS), were used to discriminate between studies. In total, 23 eligible studies with a total of 2,505 NSCLC patients were included in our meta-analysis. Our results demonstrated that decreased expression of PTEN correlated with poor overall survival in NSCLC patients and was indicative of a poor prognosis for disease-free survival and progression-free survival in patients with NSCLC.

## INTRODUCTION

Phosphatase and tensin homolog deleted on chromosome 10 (PTEN) is a protein that can modulate cell survival and cell cycle progression [1]. In healthy physiological conditions, PTEN can control smooth muscle differentiation [2], mediate angiogenesis [3], maintain Treg cell stability [4], and coordinate retinal neurogenesis [5]. However, PTEN is a tumor suppressor that is commonly down-regulated in many types of cancer [6], including non-small cell lung cancer (NSCLC) [7, 8]. Indeed, inactivation of PTEN can augment invasiveness and anchorage independent growth of NSCLC cells [9]. In addition, in some animal models, PTEN inactivation also accelerates tumorigenesis [10]. On the other hand, exogenously imported PTEN can suppress lung tumorigenesis [11]. Similarly, PTEN upregulation can inhibit NSCLC cell growth and promote partial apoptosis [12].

While many histopathology research studies reported that PTEN downregulation correlates with unfavorable survival prognosis in NSCLC patients [13–16], some studies reached the opposite conclusion [17, 18]. Therefore results in this field seem to be inconsistent. One problem is that most studies assessing the effects of PTEN expression in NSCLC were limited by small sample sizes. Here, we performed a systematic review and statistical meta-analysis of the literature to draw conclusions regarding the prognostic value of PTEN downregulation in NSCLC patients.

## RESULTS

### Study selection and characteristics

**Figure 1:**
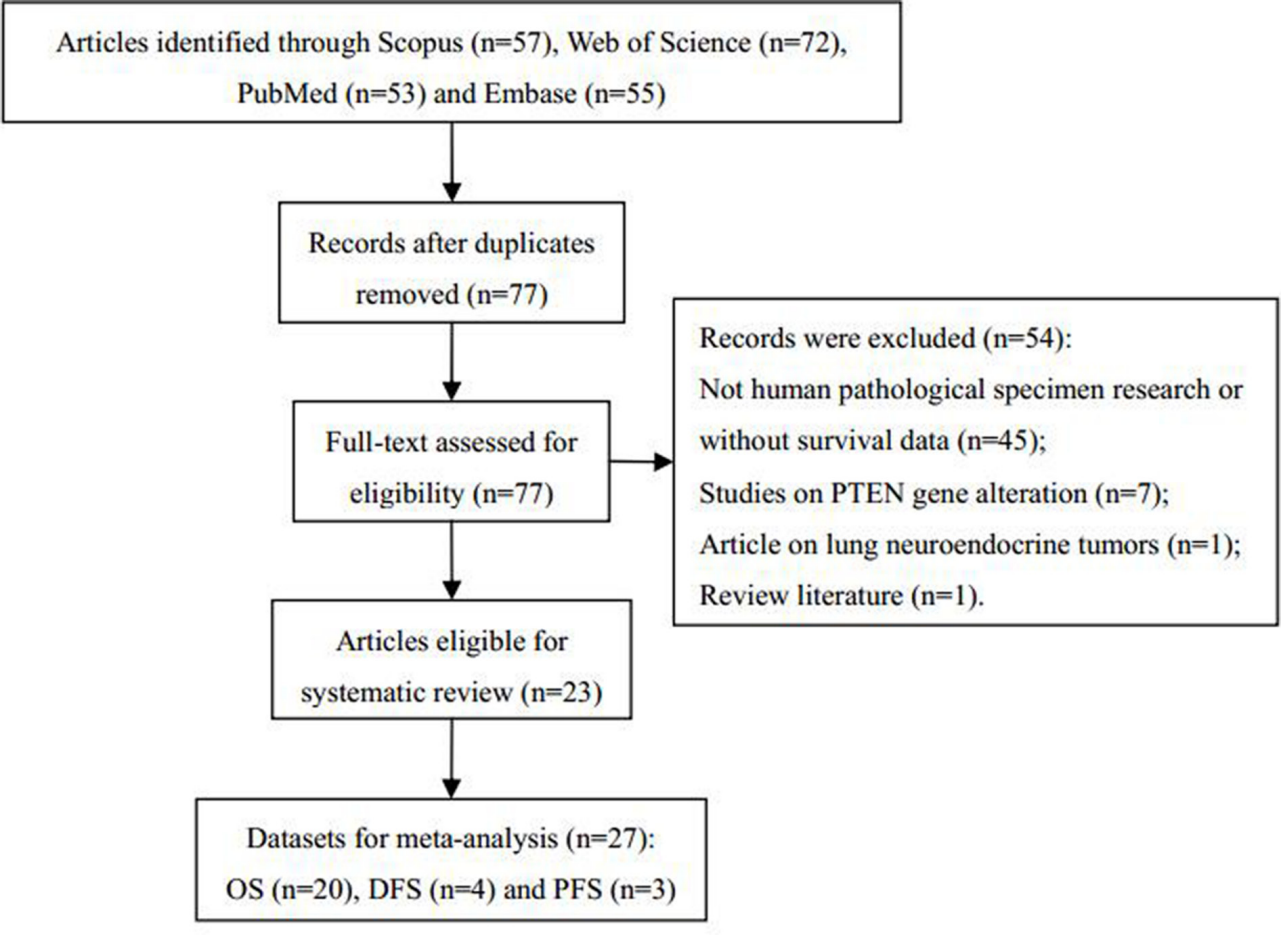
Flow diagram of the selection process in this meta-analysis OS = overall survival; DFS = disease-free survival; PFS = progression-free survival.

Our initial database search identified 237 potentially relevant records. After the duplicates were removed, 77 records remained, of which 54 were excluded because they failed to meet our inclusion criteria (see Materials and Methods). In the end, a total of 23 studies [13–35] were included in this systematic review (Figure 1). The main characteristics of these studies are summarized in Table 1 and Table S1. In total, 2,505 NSCLC patients were included in our meta-analysis. According to the different survival outcomes, patients from the 23 eligible studies were divided into 27 datasets: 20 for overall survival (OS), four for disease-free survival (DFS) and three for progression-free survival (PFS) (Table 1 and Figure 1). The Newcastle-Ottawa Quality Assessment Scale (NOS) scores of the included studies ranged from five to eight (with a mean value of 6.22), indicating that these studies were of moderate to high quality (Table S2).

**Table 1:** Main characteristics of eligible studies

First author	Year	Region	Cases	Expression	Method	Treatment	Outcome	Analysis	HR estimation	Follow-up time
Shen H [13]	2016	China	51	Protein	IHC	Surgery/ EGFR-TKI	OS	Univariate	Survival curves	The max follow-up time was 66.2 months
Wang J [14]	2015	China	92	Protein	IHC	Surgery	OS	Univariate	Survival curves	Median 28 months (range, 3 to 98 month)
Tang YA [15]	2015	Taiwan	133	Protein	IHC	Surgery	OS	Univariate	Survival curves	The max follow-up time was 126.8 months
Li XB [16]	2015	China	68	Protein	IHC	Surgery	OS	Multivariate	Reported data	Median 12.5 months (range, 3.6 to 40.6 months)
Ji Y [19]	2014	China	67	Protein	IHC	Surgery	OS	Multivariate	Reported data	The max follow-up time was 40 months
Shen H [20]	2014	China	46	Protein	IHC	Surgery/ EGFR-TKI	OS	Univariate	Survival curves	The max follow-up time was 66 months
Hu J [21]	2012	China	114	Protein	IHC	Surgery	OS	Univariate	Reported data	Median 40.10 months (range, 2.23 to 67.77 months)
Wang L [22]	2012	China	78	Protein	IHC	Surgery	PFS	Univariate	Survival curves	The max follow-up time was 24 months
An SJ [23]	2012	China	97	Protein	IHC	Surgery	OS	Univariate	Survival curves	Median 53.9 months
Shih MC [24]	2012	Taiwan	119	Protein	IHC	Surgery	OS	Univariate	Survival curves	The max follow-up time was 120 months
Shih MC [24]	2012	Taiwan	119	Protein	IHC	Surgery	DFS	Univariate	Survival curves	The max follow-up time was 120 months
Yanagawa N [25]	2012	Canada	152	Protein	IHC	Surgery	DFS	Multivariate	Reported data	Median 28.56 months (range, 0.84 to 71.64 months)
Yoo SB [26]	2011	Korea	288	Protein	IHC	Surgery/ Chemotherapy/ Radiation therapy/ EGFR-TKI	PFS	Multivariate	Reported data	Median 44 months (range, 1 to 84 months)
Cetin Z [27]	2010	Turkey	50	Protein	WB	Surgery	OS	Univariate	Survival curves	The max follow-up time was 34.2 months
Buckingham L [17]	2010	USA	132	Protein	IHC	Surgery	OS	Univariate	Survival curves	The max follow-up time was 60.2 months
Wang C [28]	2009	China	249	Protein	IHC	Surgery	OS	Multivariate	Reported data	The max follow-up time was 83 months
Inamura K [18]	2007	Japan	115	mRNA	PCR	Surgery	OS	Univariate	Survival curves	The max follow-up time was 109.7 months
Zheng H [29]	2007	Japan	143	Protein	IHC	NR	OS	Univariate	Survival curves	Mean 20.6 months (range, 1 to 144 months)
Lim WT [30]	2007	USA	25	Protein	IHC	Gefitinib	PFS	Univariate	Survival curves	The max follow-up time was 100.8 months
Lim WT [30]	2007	USA	25	Protein	IHC	Gefitinib	OS	Univariate	Survival curves	The max follow-up time was 100.8 months
Endoh H [31]	2006	Japan	78	mRNA	PCR	Surgery/ Chemotherapy/ Gefitinib	OS	Univariate	Reported data	Median 4 months (range, 0.8 to 31.3 months)
Tang JM [32]	2006	China	102	Protein	IHC	Surgery	OS	Multivariate	Reported data	Median 25.5 months (range, 3 to 60 months)
Ferraro B [33]	2005	USA	125	mRNA	PCR	Surgery	DFS	Univariate	Survival curves	Median 101 months (range, 39 to 161 months)
Ferraro B [33]	2005	USA	125	mRNA	PCR	Surgery	OS	Univariate	Survival curves	Median 101 months (range, 39 to 161 months)
Bepler G [34]	2004	USA	77	mRNA	PCR	Surgery	OS	Multivariate	Reported data	Median 39.7 months (range, 2.0 to 106.1 months)
Bepler G [34]	2004	USA	77	mRNA	PCR	Surgery	DFS	Univariate	Survival curves	Median 39.7 months (range, 2.0 to 106.1 months)
Goncharuk VN [35]	2004	USA	104	Protein	IHC	Surgery	OS	Univariate	Survival curves	Mean 52 months (range, 5 to 127 months)

IHC: Immunohistochemistry; WB: Western blot; PCR: Polymerase chain reaction; NR: No report; EGFR-TKI: Epidermal growth factor receptor-tyrosine kinase inhibitor; OS: Overall survival; DFS: disease-free survival; PFS: progression-free survival; HR: Hazard ratio.

### Meta-analysis of OS

**Figure 2:**
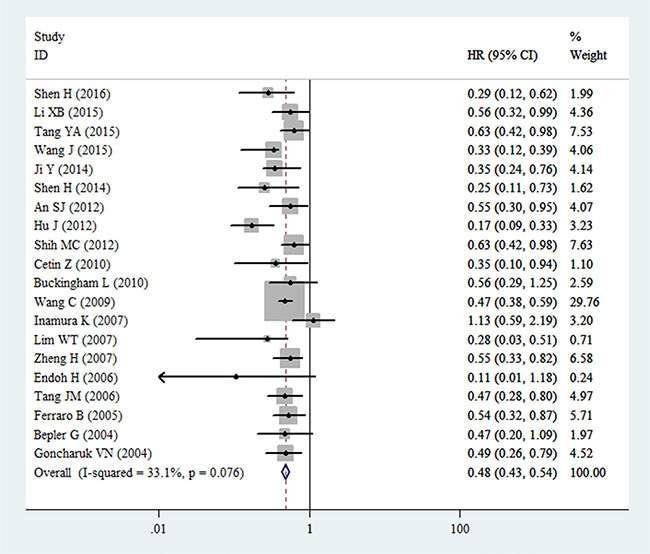
Forest plot for the relationships between decreased expression of PTEN and OS in patients with NSCLC HR = hazard ratio; CI = confidence interval.

**Figure 3:**
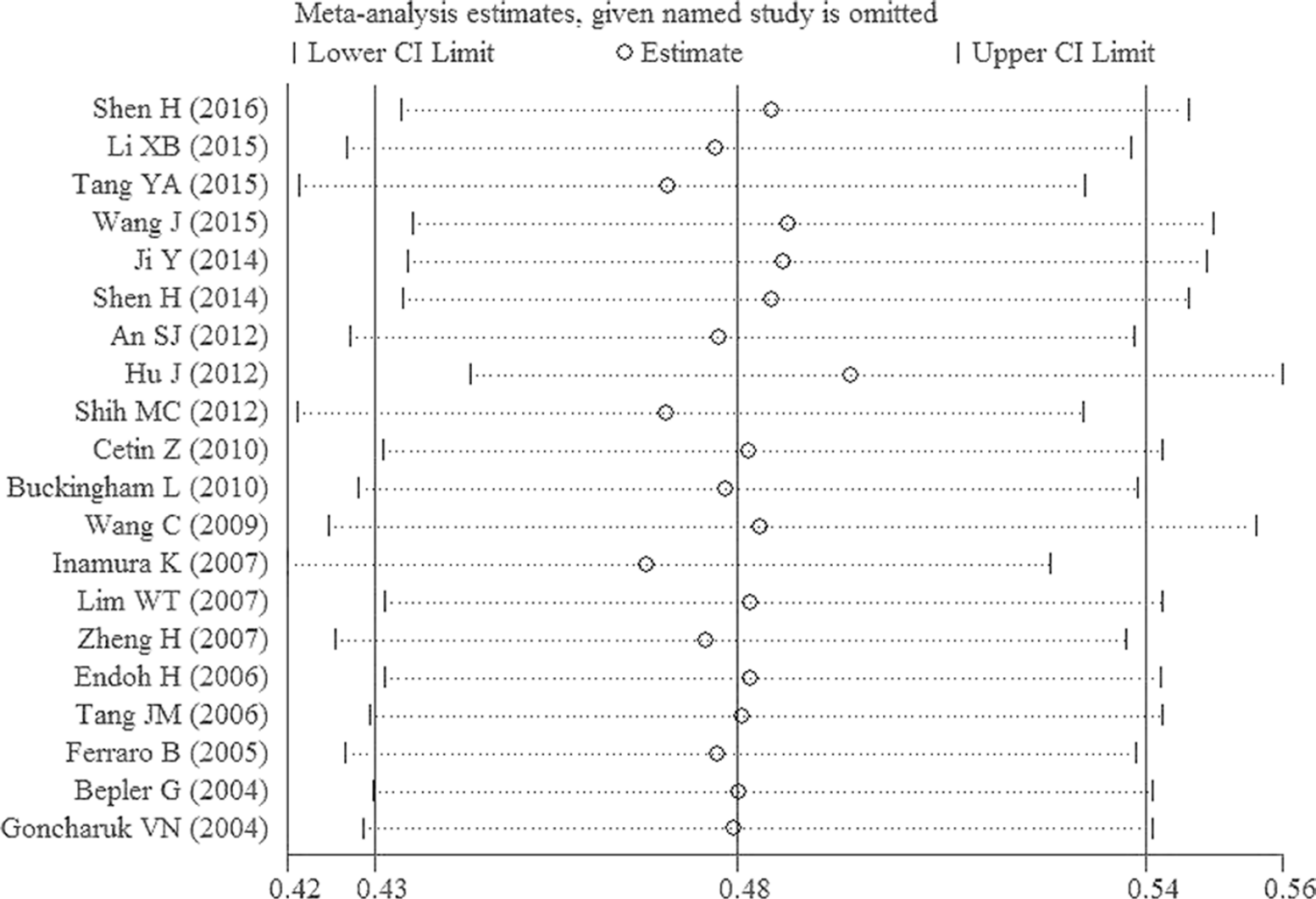
Sensitivity analysis on the relationship between decreased expression of PTEN and OS in patients with NSCLC CI = confidence interval.

The pooled result from 20 datasets revealed significant association between decreased PTEN expression and poor OS in patients with NSCLC (HR = 0.48, 95% CI: 0.43–0.54, *P* < 0.001) (Figure 2). Meanwhile, no obvious heterogeneity was found (*I^2^* = 33.1%, *P* = 0.076). By successively omitting each study, sensitivity analysis was performed to evaluate the impact of every study on the pooled HR. Results showed that the pooled HRs were no different with the exclusion of any individual study, implying that the result of the meta-analysis of OS is stable (Figure 3).

Subgroup analyses were performed depending on protein and mRNA expression, type of analysis (univariate vs. multivariate), population (Asian vs. non Asian), number of cases (less than 100 vs. more than 100) and year (after 2010 vs. before 2010). Results showed that decreased expression of PTEN protein had an unfavorable prognostic value in NSCLC patients (HR = 0.46, 95% CI: 0.39–0.53, *P* < 0.001). However, although decreased expression of PTEN mRNA also correlated with poor OS in patients with NSCLC, its value did not reach statistical significance (HR = 0.60, 95% CI: 0.34–1.07, *P* = 0.084) (Figure S1, Table 2). The results from analyzing both the univariate and multivariate analysis subgroups indicated that decreased expression of PTEN was associated with poor OS in NSCLC patients (HR = 0.47, 95% CI: 0.37–0.59, *P* < 0.001; HR = 0.47, 95% CI: 0.39–0.56, *P* < 0.001, respectively) (Figure S2, Table 2). In addition, the pooled results of other subgroup analyses showed a similar prognostic value for decreased expression of PTEN (Figures S3–S5, Table 2).

**Table 2:** Meta-analysis results for the association between decreased expression of PTEN and OS in patients with NSCLC

Categories	Subgroups	Number of datasets	HR (95% CI)	*P*-Value	Heterogeneity	
					*I*^2^	*P*-Value
**All ^F^**		20	0.483 (0.429–0.543)	< 0.001	33.1%	0.076
**Expression ^R^**	Protein	16	0.456 (0.389–0.535)	< 0.001	24.9%	0.173
	mRNA	4	0.604 (0.340–1.070)	0.084	50.4%	0.110
**Analysis ^R^**	Univariate	15	0.466 (0.368–0.589)	< 0.001	47.8%	0.020
	Multivariate	5	0.469 (0.393–0.558)	< 0.001	0.0%	0.852
**Population ^R^**	Asian	15	0.461 (0.376–0.566)	< 0.001	49.0%	0.017
	Non Asian	5	0.502 (0.372–0.677)	< 0.001	0.0%	0.929
**Cases ^R^**	Less than 100	10	0.396 (0.311–0.503)	< 0.001	0.0%	0.721
	More than 100	10	0.523 (0.421–0.649)	< 0.001	51.9%	0.028
**Year ^R^**	After 2010	9	0.412 (0.307–0.554)	< 0.001	56.0%	0.020
	Before 2010	11	0.506 (0.436–0.589)	< 0.001	0.0%	0.514

F For fixed-effects model;

R for random-effects model;

HR: Hazard ratio; CI: Confidence intervals.

### Meta-analysis of DFS/PFS

**Figure 4:**
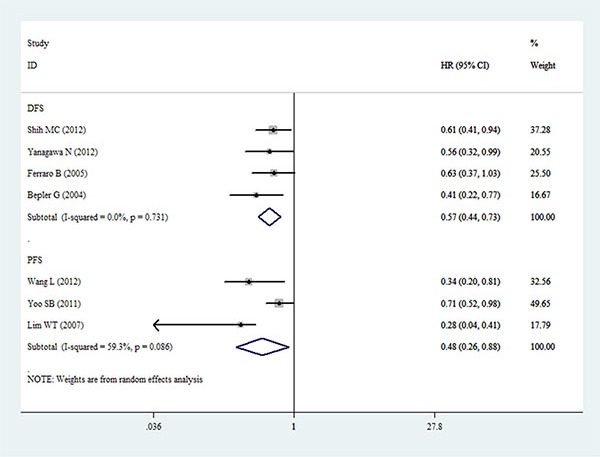
Forest plot for the relationships between decreased expression of PTEN and DFS/PFS in patients with NSCLC DFS = disease-free survival; PFS = progression-free survival; HR = hazard ratio.

The pooled result from four datasets of DFS revealed that decreased expression of PTEN was associated with unfavorable DFS in patients with NSCLC (HR = 0.57, 95% CI: 0.44–0.73, *P* < 0.001) (Figure 4). Similarly, the pooled result from three datasets of PFS showed that decreased expression of PTEN also had an unfavorable prognostic value for PFS in NSCLC patients (HR = 0.48, 95% CI: 0.26–0.88, *P* = 0.018) (Figure 4).

### Publication bias

Both Begg's and Egger's test were used to evaluate the publication bias of the meta-analysis of OS. The result of Egger's test showed no publication bias (*P* = 0.169) while Begg's test indicated that publication bias might exist (*P* = 0.012). We used the trim-and-fill method to estimate the influence of potential publication bias. As a result, three theoretical studies were added in the meta-analysis of OS (Figure S6). The recalculated pooled result did not change significantly (HR = 0.51, 95% CI: 0.45–0.57, *P* < 0.001), indicating the stability of the result.

## DISCUSSION

All 23 studies we analyzed met specific inclusion criteria and had moderate to high quality according to their NOS scores. Overall, 2,505 NSCLC patients were included and the survival data were organized based on overall survival (OS), disease-free survival (DFS) and progression-free survival (PFS). The combined results demonstrated that decreased expression of PTEN correlated with poor OS in NSCLC patients. What's more, we found that decreased expression of PTEN also indicated a poor prognosis for DFS and PFS in patients with NSCLC.

In our meta-analysis, the results from analyzing both the univariate and multivariate subgroups indicated that decreased expression of PTEN was associated with poor OS in patients with NSCLC. However, multivariate analysis ruled out the compounding effects from other clinicopathological factors such as sex, age, tumor size, nodal status and stage, among others [16, 19, 28, 32, 34]. Thus, according to the pooled result from analyzing the multivariate analysis subgroup, expression of PTEN may be considered to be an independent factor of poor prognosis in NSCLC patients. While decreased expression of PTEN protein correlated with poor OS in patients with NSCLC, decreased expression of PTEN mRNA did not. This is not surprising since mRNA levels from an expressed gene do not usually predict the corresponding protein levels [36, 37]. Immunohistochemistry is used as a complementary diagnostic method in 95% of cancer cases [38] because it can benefit surgical and therapeutic decisions at a low cost. Therefore, we recommend clinicians to use PTEN protein expression detected by immunohistochemistry as a prognostic factor to treat NSCLC patients.

Prior to our study, two meta-analyses had been performed to evaluate the association between PTEN expression and the survival of cancer patients. According to the combined results of nine original studies, Chen J. *et al.* concluded that reduced PTEN expression correlated with poor OS in patients with gastric cancer [39]. On the other hand, the meta-analysis of 14 original studies by Cai J. *et al.* found that the expression of PTEN had no prognostic value for OS in patients with epithelial ovarian cancer [40]. However, in our current meta-analysis, the combined results from 20 original studies showed that decreased expression of PTEN was indeed associated with poor OS in NSCLC patients. These results suggest that the correlation between PTEN expression and OS in cancer patients may differ depending on cancer type.

Among our eligible studies, four [24, 25, 33, 34] reported statistics for DFS and three [22, 26, 30] for PFS. Most of the patients in such studies had undergone surgical resection. Yoo S.B. *et al.* [26] reported a cohort of 288 consecutive NSCLC patients who underwent surgical resection and further received post operative adjuvant chemotherapy and radiation therapy. Among them, some patients received additional EGFR-TKIs, such as gefitinib or erlotinib [26]. In addition, Lim W.T. *et al.* [30] also reported a cohort of NSCLC patients that had only been treated with gefitinib. Overall, our results combining data from both of the aforementioned studies revealed that decreased expression of PTEN was associated with a shorter DFS as well as a shorter PFS. This means that in addition to the prognostic value of decreased PTEN expression on OS, PTEN expression levels can also inform on the effect of treatment for patients with NSCLC.

Tumor suppression by PTEN depends on its negative regulation of the phosphatidylinositol 3-kinase-Akt-mammalian target of rapamycin (PI3K-Akt-mTOR) signaling pathway [41]. Thus, PTEN is regarded as the controller of this pathway [42]. Consequently, when the expression of PTEN is decreased, either inhibiting PI3K or controlling the PI3K-Akt-mTOR pathway in other ways can supplement PTEN's tumor suppression [43–46]. Therefore, we consider that NSCLC patients with decreased expression of PTEN suffer from a subtype of lung cancer and might benefit from individualized treatment plans.

Several limitations should be noticed in our meta-analysis. One of the main limitations is the potential publication bias, stemming from published results being predominantly positive, since all of our included studies were retrospectively designed. Furthermore, patient populations in our study were limited, as patients came only from Asia and North America. Additionally, some of the survival data we used were extracted from survival curves, which may introduce subjective bias. Finally, the studies reporting DFS and PFS were few in number. Therefore, further studies without these biases might strengthen our conclusions. Nonetheless, our meta-analyses showed that decreased expression of PTEN predicted a shorter OS, DFS and PFS in the populations of patients with NSCLC analyzed.

## MATERIALS AND METHODS

### Search strategy

We systematically searched in the online Scopus, Web of Science, PubMed and Embase databases (updated until May 22, 2016) with the restrictions of English language and original article. Two investigators (Jian Xiao and Bi-Xiu He) independently screened all titles and abstracts to identify eligible studies. The search terms used included “PTEN”, “NSCLC” and derivative terms (File S1). Manual searches of the included studies and published reviews were also conducted.

### Study selection

In this meta-analysis, studies were selected according to the following criteria: (1) original studies measured the PTEN expression in patients with NSCLC; (2) reported the correlation between PTEN expression and patient survival; (3) reported the hazard ratios (HRs), and their corresponding 95% confidence intervals (CIs) could be obtained. Of note, we would include the most complete report if the same authors reported repeated results. However, unpublished studies, meeting abstracts, case reports, comments, letters, meta-analyses and literature reviews were excluded.

### Data extraction

Two raters independently extracted the primary information using a standardized form and disagreements were discussed until a consensus was reached. Except for the HRs and their corresponding 95% CIs, the following information categories were also extracted: first author, year of publication, region of population, number of cases, PTEN expression, test method, survival outcome, analysis method, HR estimation, follow-up time and cut-off value. When both multivariate and univariate analyses of the survival results were reported, we extracted the HRs and their corresponding 95% CIs from multivariate analyses. However, when HR and its corresponding 95% CI were not reported as calculated data, they were estimated using the previously reported methods [47, 48], according to other relevant information (e.g., survival curves).

### Quality assessment

The Newcastle-Ottawa Quality Assessment Scale (NOS) was used to assess the quality of the included studies and was conducted by two independent investigators. Disagreements were resolved by discussion. In brief, NOS is comprised of three parameters of quality: selection, comparability, and outcome assessment. Furthermore, each study received a total score between 0 and 9, with a NOS score of 7 or above considered as high quality and a NOS score of 3 or below considered as low quality [48–50]. Details of the quality assessment of included studies are provided in File S2 and Table S2.

### Statistical analysis

We used Stata 12.0 (StataCorp LP) and R software (https://www.r-project.org/) to perform all statistical analyses. HRs and their corresponding 95% CIs were calculated for all of the survival outcomes. When the pooled HR was lower than 1, we considered that the decreased expression of PTEN was associated with unfavorable survival in patients with NSCLC. Heterogeneity analysis was conducted using Cochran's *Q* test and Higgins' I-squared statistic and Heterogeneity was defined either as *I^2^* > 50% or *P* < 0.05. A random-effects model was used when heterogeneity was present; otherwise, the fixed-effects model was used. The stability of the pooled HR results was assessed by the sensitivity analysis. Publication bias was evaluated using Begg's and Egger's tests. If publication bias existed, we applied the trim-and-fill method. For all of our results, *P* < 0.05 (two-tailed) was defined to be statistically significant.

## SUPPLEMENTARY MATERIALS FIGURES AND TABLES



## References

[1] Sun H, Lesche R, Li DM, Liliental J, Zhang H, Gao J, Gavrilova N, Mueller B, Liu X, Wu H (1999). PTEN modulates cell cycle progression and cell survival by regulating phosphatidylinositol 3 4 5-trisphosphate and Akt/protein kinase B signaling pathway. Proc Natl Acad Sci USA.

[2] Horita H, Wysoczynski CL, Walker LA, Moulton KS, Li M, Ostriker A, Tucker R, McKinsey TA, Churchill ME, Nemenoff RA, Weiser-Evans MC (2016). Nuclear PTEN functions as an essential regulator of SRF-dependent transcription to control smooth muscle differentiation. Nat Commun.

[3] Serra H, Chivite I, Angulo-Urarte A, Soler A, Sutherland JD, Arruabarrena-Aristorena A, Ragab A, Lim R, Malumbres M, Fruttiger M, Potente M, Serrano M, Fabra A (2015). PTEN mediates Notch-dependent stalk cell arrest in angiogenesis. Nat Commun.

[4] Shrestha S, Yang K, Guy C, Vogel P, Neale G, Chi H (2015). Treg cells require the phosphatase PTEN to restrain TH1 and TFH cell responses. Nat Immunol.

[5] Jo HS, Kang KH, Joe CO, Kim JW (2012). Pten coordinates retinal neurogenesis by regulating Notch signalling. EMBO J.

[6] Song MS, Salmena L, Pandolfi PP (2012). The functions and regulation of the PTEN tumour suppressor. Nat Rev Mol Cell Biol.

[7] Maeda M, Murakami Y, Watari K, Kuwano M, Izumi H, Ono M (2015). CpG hypermethylation contributes to decreased expression of PTEN during acquired resistance to gefitinib in human lung cancer cell lines. Lung Cancer.

[8] Noro R, Gemma A, Miyanaga A, Kosaihira S, Minegishi Y, Nara M, Kokubo Y, Seike M, Kataoka K, Matsuda K, Okano T, Yoshimura A, Kudoh S (2007). PTEN inactivation in lung cancer cells and the effect of its recovery on treatment with epidermal growth factor receptor tyrosine kinase inhibitors. Int J Oncol.

[9] Akca H, Demiray A, Tokgun O, Yokota J (2011). Invasiveness and anchorage independent growth ability augmented by PTEN inactivation through the PI3K/AKT/NFkB pathway in lung cancer cells. Lung Cancer.

[10] Iwanaga K, Yang Y, Raso MG, Ma L, Hanna AE, Thilaganathan N, Moghaddam S, Evans CM, Li H, Cai WW, Sato M, Minna JD, Wu H (2008). Pten inactivation accelerates oncogenic K-ras-initiated tumorigenesis in a mouse model of lung cancer. Cancer Res.

[11] Jin H, Xu CX, Kim HW, Chung YS, Shin JY, Chang SH, Park SJ, Lee ES, Hwang SK, Kwon JT, Minai-Tehrani A, Woo M, Noh MS (2008). Urocanic acid-modified chitosan-mediated PTEN delivery via aerosol suppressed lung tumorigenesis in K-ras(LA1) mice. Cancer Gene Ther.

[12] Bi L, Chen J, Yuan X, Jiang Z, Chen W (2013). Salvianolic acid A positively regulates PTEN protein level and inhibits growth of A549 lung cancer cells. Biomed Rep.

[13] Shen H, Guan D, Shen J, Wang M, Chen X, Xu T, Liu L, Shu Y (2016). TGF-beta1 induces erlotinib resistance in non-small cell lung cancer by down-regulating PTEN. Biomed Pharmacother.

[14] Wang J, Chen H, Liao Y, Chen N, Liu T, Zhang H, Zhang H (2015). Expression and clinical evidence of miR-494 and PTEN in non-small cell lung cancer. Tumour Biol.

[15] Tang YA, Chen CH, Sun HS, Cheng CP, Tseng VS, Hsu HS, Su WC, Lai WW, Wang YC (2015). Global Oct4 target gene analysis reveals novel downstream PTEN and TNC genes required for drug-resistance and metastasis in lung cancer. Nucleic Acids Res.

[16] Li XB, Yang Y, Zhang HQ, Yue WT, Zhang TM, Lu BH, Li J, Liu Z, Wang QH, Gao Y, Hu AM, Zhang HM, Shi HL (2015). High levels of phosphatase and tensin homolog expression predict favorable prognosis in patients with non-small cell lung cancer. Eur Rev Med Pharmacol Sci.

[17] Buckingham L, Penfield Faber L, Kim A, Liptay M, Barger C, Basu S, Fidler M, Walters K, Bonomi P, Coon J (2010). PTEN RASSF1 and DAPK site-specific hypermethylation and outcome in surgically treated stage I and II nonsmall cell lung cancer patients. Int J Cancer.

[18] Inamura K, Togashi Y, Nomura K, Ninomiya H, Hiramatsu M, Okui M, Satoh Y, Okumura S, Nakagawa K, Tsuchiya E, Ishikawa Y (2007). Up-regulation of PTEN at the transcriptional level is an adverse prognostic factor in female lung adenocarcinomas. Lung Cancer.

[19] Ji Y, Zheng M, Ye S, Chen J, Chen Y (2014). PTEN and Ki67 expression is associated with clinicopathologic features of non-small cell lung cancer. J Biomed Res.

[20] Shen H, Zhu F, Liu J, Xu T, Pei D, Wang R, Qian Y, Li Q, Wang L, Shi Z, Zheng J, Chen Q, Jiang B (2014). Alteration in Mir-21/PTEN expression modulates gefitinib resistance in non-small cell lung cancer. PLoS One.

[21] Hu J, Liu YL, Piao SL, Yang DD, Yang YM, Cai L (2012). Expression patterns of USP22 and potential targets BMI-1 PTEN p-AKT in non-small-cell lung cancer. Lung Cancer.

[22] Wang L, Yue W, Zhang L, Zhao X, Wang Y, Xu S (2012). mTOR and PTEN expression in non-small cell lung cancer: analysis by real-time fluorescence quantitative polymerase chain reaction and immunohistochemistry. Surg Today.

[23] An SJ, Lin QX, Chen ZH, Su J, Cheng H, Xie Z, Zhang XC, Zhou HY, Huang Y, Chen SL, Guo WB, Wu YL (2012). Combinations of laminin 5 with PTEN p-EGFR and p-Akt define a group of distinct molecular subsets indicative of poor prognosis in patients with non-small cell lung cancer. Exp Ther Med.

[24] Shih MC, Chen JY, Wu YC, Jan YH, Yang BM, Lu PJ, Cheng HC, Huang MS, Yang CJ, Hsiao M, Lai JM (2012). TOPK/PBK promotes cell migration via modulation of the PI3K/PTEN/AKT pathway and is associated with poor prognosis in lung cancer. Oncogene.

[25] Yanagawa N, Leduc C, Kohler D, Saieg MA, John T, Sykes J, Yoshimoto M, Pintilie M, Squire J, Shepherd FA, Tsao MS (2012). Loss of phosphatase and tensin homolog protein expression is an independent poor prognostic marker in lung adenocarcinoma. J Thorac Oncol.

[26] Yoo SB, Xu X, Lee HJ, Jheon S, Lee C-T, Choe G, Chung J–H (2011). Loss of PTEN Expression is an Independent Poor Prognostic Factor in Non-small Cell Lung Cancer. Korean Journal of Pathology.

[27] Cetin Z, Ozbilim G, Erdogan A, Luleci G, Karauzum SB (2010). Evaluation of PTEN and Mcl-1 expressions in NSCLC expressing wild-type or mutated EGFR. Medical Oncology.

[28] Wang C, Yang R, Yue D, Zhang Z (2009). Expression of FAK and PTEN in bronchioloalveolar carcinoma and lung adenocarcinoma. Lung.

[29] Zheng H, Tsuneyama K, Takahashi H, Miwa S, Nomoto K, Saito H, Masuda S, Takano Y (2007). Expression of PTEN and FHIT is involved in regulating the balance between apoptosis and proliferation in lung carcinomas. Anticancer Res.

[30] Lim WT, Zhang WH, Miller CR, Watters JW, Gao F, Viswanathan A, Govindan R, McLeod HL (2007). PTEN and phosphorylated AKT expression and prognosis in early- and late-stage non-small cell lung cancer. Oncol Rep.

[31] Endoh H, Yatabe Y, Kosaka T, Kuwano H, Mitsudomi T (2006). PTEN and PIK3CA expression is associated with prolonged survival after gefitinib treatment in EGFR-mutated lung cancer patients. J Thorac Oncol.

[32] Tang JM, He QY, Guo RX, Chang XJ (2006). Phosphorylated Akt overexpression and loss of PTEN expression in non-small cell lung cancer confers poor prognosis. Lung Cancer.

[33] Ferraro B, Bepler G, Sharma S, Cantor A, Haura EB (2005). EGR1 predicts PTEN and survival in patients with non-small-cell lung cancer. J Clin Oncol.

[34] Bepler G, Sharma S, Cantor A, Gautam A, Haura E, Simon G, Sharma A, Sommers E, Robinson L (2004). RRM1 and PTEN as prognostic parameters for overall and disease-free survival in patients with non-small-cell lung cancer. J Clin Oncol.

[35] Goncharuk VN, del-Rosario A, Kren L, Anwar S, Sheehan CE, Carlson JA, Ross JS (2004). Co-downregulation of PTEN KAI-1 and nm23-H1 tumor/metastasis suppressor proteins in non-small cell lung cancer. Ann Diagn Pathol.

[36] Maier T, Guell M, Serrano L (2009). Correlation of mRNA and protein in complex biological samples. FEBS Lett.

[37] Laurent JM, Vogel C, Kwon T, Craig SA, Boutz DR, Huse HK, Nozue K, Walia H, Whiteley M, Ronald PC, Marcotte EM (2010). Protein abundances are more conserved than mRNA abundances across diverse taxa. Proteomics.

[38] Matos LL, Trufelli DC, de Matos MG, da Silva Pinhal MA (2010). Immunohistochemistry as an important tool in biomarkers detection and clinical practice. Biomark Insights.

[39] Chen J, Li T, Liu Q, Jiao H, Yang W, Liu X, Huo Z (2014). Clinical and prognostic significance of HIF-1alpha PTEN CD44v6 and survivin for gastric cancer: a meta-analysis. PLoS One.

[40] Cai J, Xu L, Tang H, Yang Q, Yi X, Fang Y, Zhu Y, Wang Z (2014). The role of the PTEN/PI3K/Akt pathway on prognosis in epithelial ovarian cancer: a meta-analysis. Oncologist.

[41] Hollander MC, Blumenthal GM, Dennis PA (2011). PTEN loss in the continuum of common cancers rare syndromes and mouse models. Nat Rev Cancer.

[42] Georgescu MM (2010). PTEN Tumor Suppressor Network in PI3K-Akt Pathway Control. Genes Cancer.

[43] Barlaam B, Cosulich S, Degorce S, Fitzek M, Green S, Hancox U, Lambert-van der Brempt C, Lohmann JJ, Maudet M, Morgentin R, Pasquet MJ, Peru A, Ple P (2015). Discovery of (R)-8-(1-(3 5-difluorophenylamino)ethyl)-N N-dimethyl-2-morpholino-4-oxo-4H-chrom ene-6-carboxamide (AZD8186): a potent and selective inhibitor of PI3Kbeta and PI3Kdelta for the treatment of PTEN-deficient cancers. J Med Chem.

[44] Barlaam B, Cosulich S, Degorce S, Fitzek M, Green S, Hancox U, Lambert-van der Brempt C, Lohmann JJ, Maudet M, Morgentin R, Peru A, Ple P, Saleh T (2016). Discovery of a series of 8-(2 3-dihydro-1 4-benzoxazin-4-ylmethyl)-2-morpholino-4-oxo-chromene-6-carboxami des as PI3Kbeta/delta inhibitors for the treatment of PTEN-deficient tumours. Bioorg Med Chem Lett.

[45] Yamamoto Y, De Velasco MA, Kura Y, Nozawa M, Hatanaka Y, Oki T, Ozeki T, Shimizu N, Minami T, Yoshimura K, Yoshikawa K, Nishio K, Uemura H (2015). Evaluation of *in vivo* responses of sorafenib therapy in a preclinical mouse model of PTEN-deficient of prostate cancer. J Transl Med.

[46] Zhang W, Haines BB, Efferson C, Zhu J, Ware C, Kunii K, Tammam J, Angagaw M, Hinton MC, Keilhack H, Paweletz CP, Zhang T, Winter C (2012). Evidence of mTOR Activation by an AKT-Independent Mechanism Provides Support for the Combined Treatment of PTEN-Deficient Prostate Tumors with mTOR and AKT Inhibitors. Transl Oncol.

[47] Krieg A, Riemer JC, Telan LA, Gabbert HE, Knoefel WT (2015). CXCR4—A Prognostic and Clinicopathological Biomarker for Pancreatic Ductal Adenocarcinoma: A Meta-Analysis. PLoS One.

[48] Xiao J, Zou Y, Chen X, Gao Y, Xie M, Lu X, Li W, He B, He S, You S, Chen Q (2016). The Prognostic Value of Decreased LKB1 in Solid Tumors: A Meta-Analysis. PLoS One.

[49] Gu XB, Tian T, Tian XJ, Zhang XJ (2015). Prognostic significance of neutrophil-to-lymphocyte ratio in non-small cell lung cancer: a meta-analysis. Sci Rep.

[50] Dahal K, Kunwar S, Rijal J, Schulman P, Lee J (2016). Stroke Major Bleeding and Mortality Outcomes in Warfarin Users With Atrial Fibrillation and Chronic Kidney Disease: A Meta-Analysis of Observational Studies. Chest.

