# Supplementary material for: PTEN expression is a prognostic marker for patients with non-small cell lung cancer: a systematic review and meta-analysis of the literature
Source: Oncotarget. 2016 Aug 5;7(36):57832–40. doi: 10.18632/oncotarget.11068 (PMC5295393; doi:10.18632/oncotarget.11068)
Supplement: Supplementary file 1 [file oncotarget-07-57832-s001.pdf]

PTEN expression is a prognostic marker for patients with non-small cell lung cancer: a systematic review and meta-analysis of the literature

Supplementary Materials

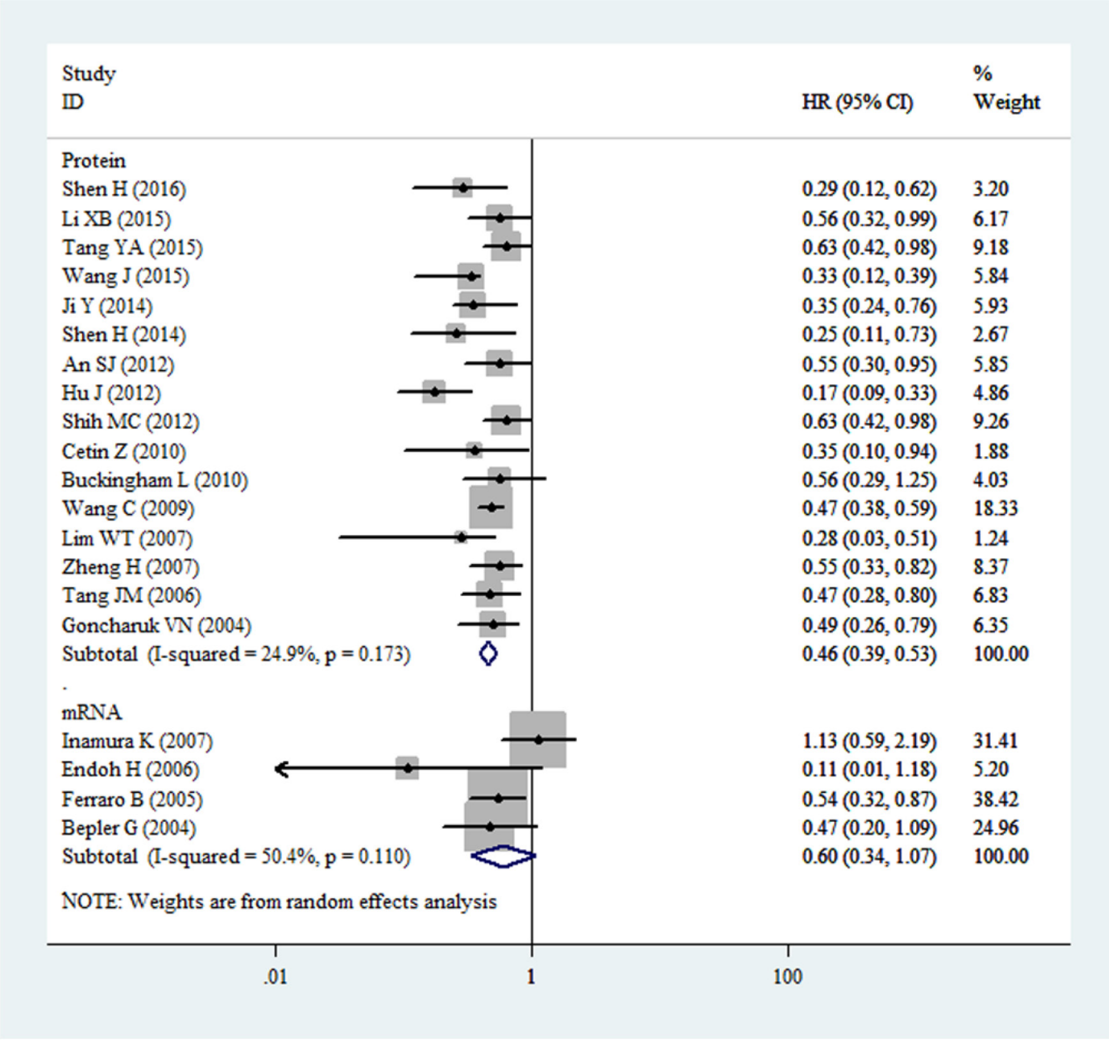

Supplementary Figure S1: Forest plot for the subgroup analysis according to different expression. HR = hazard ratio; CI = confidence interval.

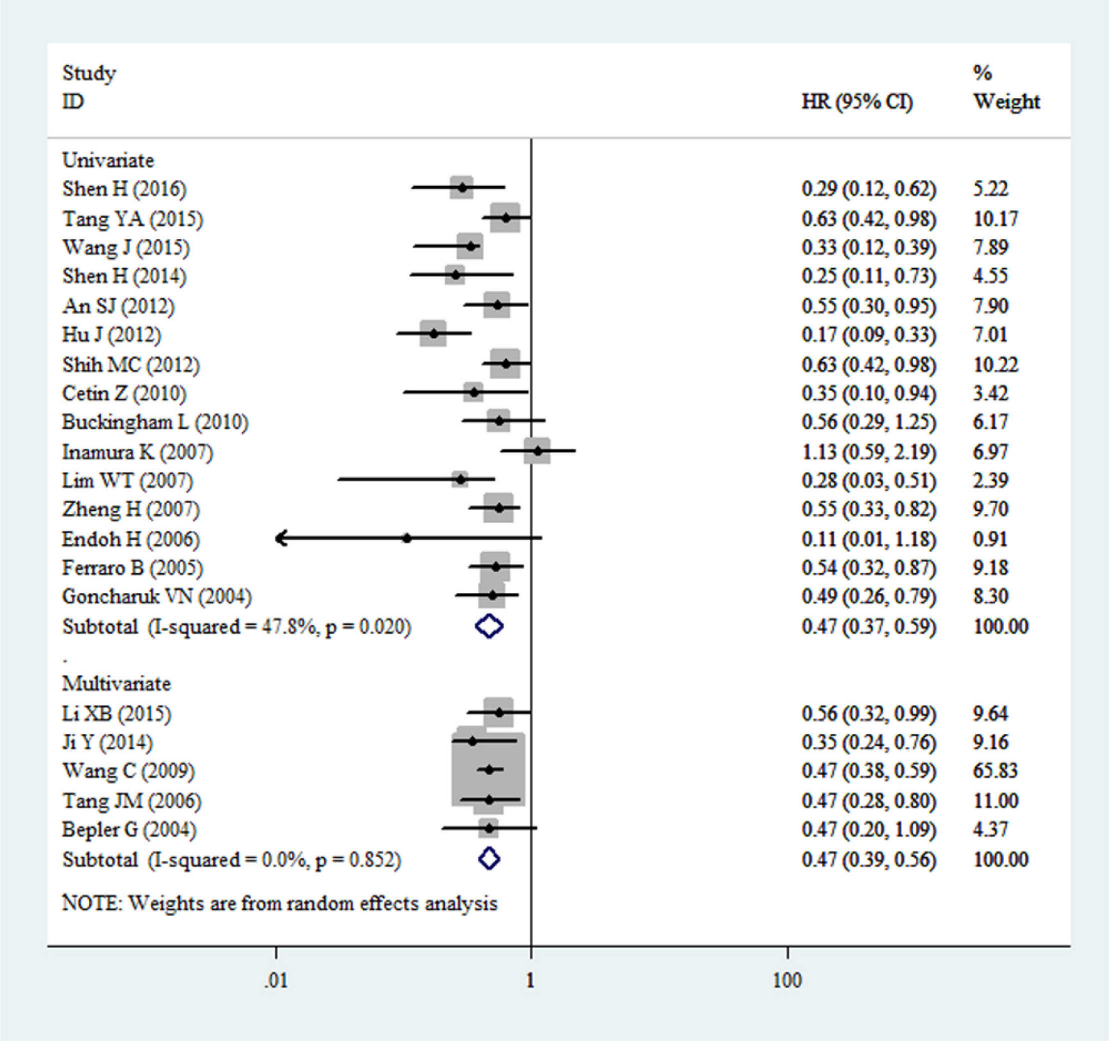

Supplementary Figure S2: Forest plot for the subgroup analysis according to different analysis method. HR = hazard ratio; CI = confidence interval.

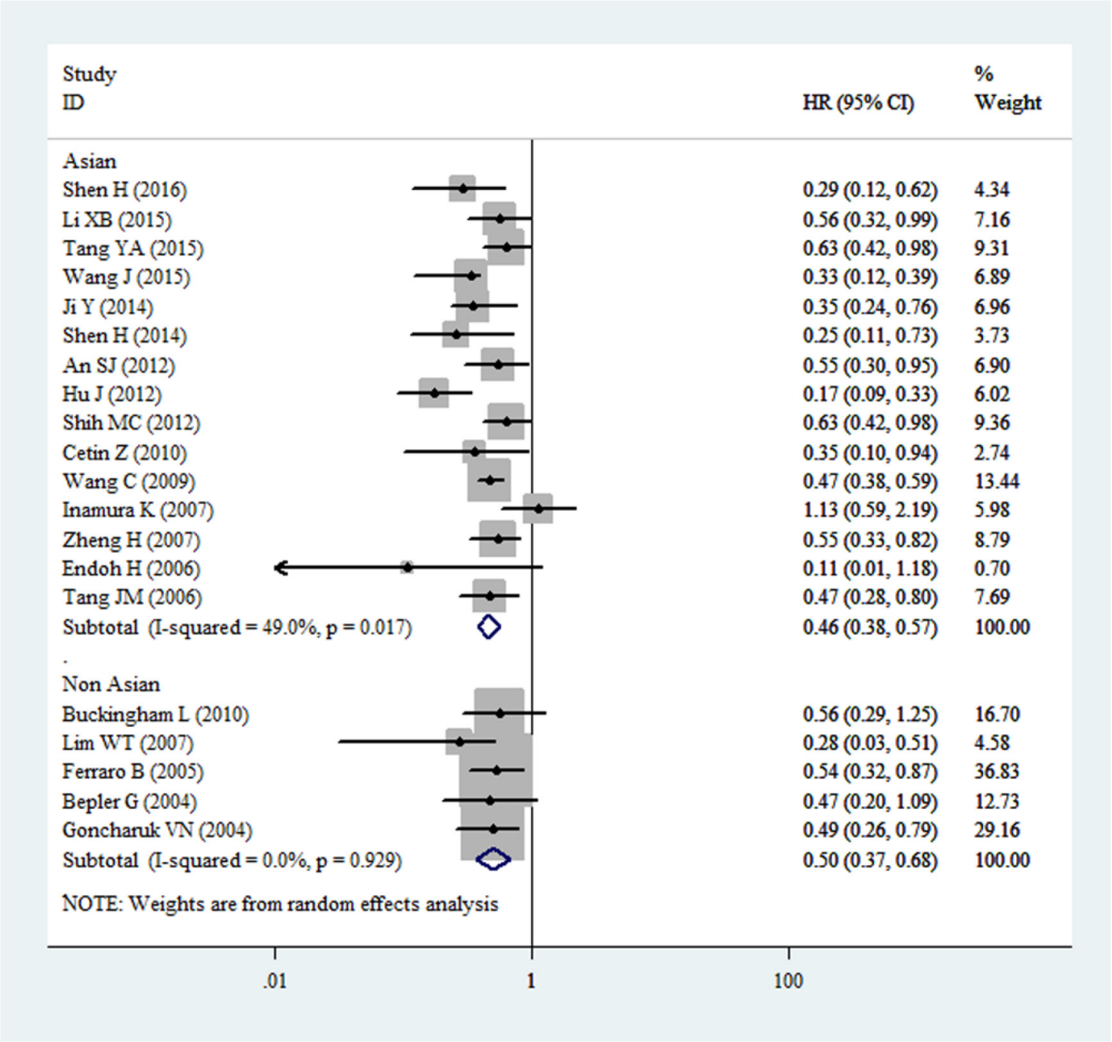

Supplementary Figure S3: Forest plot for the subgroup analysis according to different population. HR = hazard ratio; CI = confidence interval.

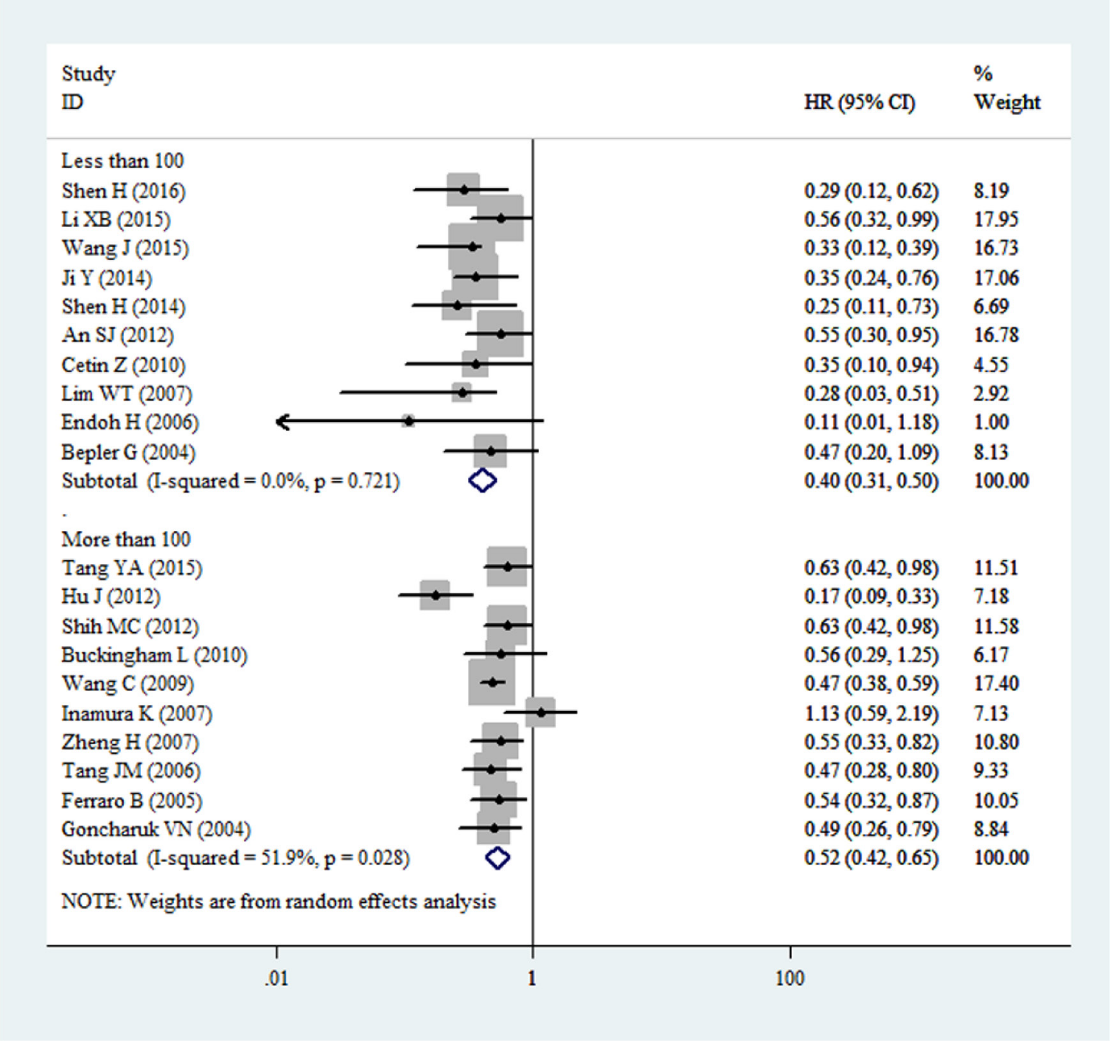

Supplementary Figure S4: Forest plot for the subgroup analysis of cases. HR = hazard ratio; CI = confidence interval.

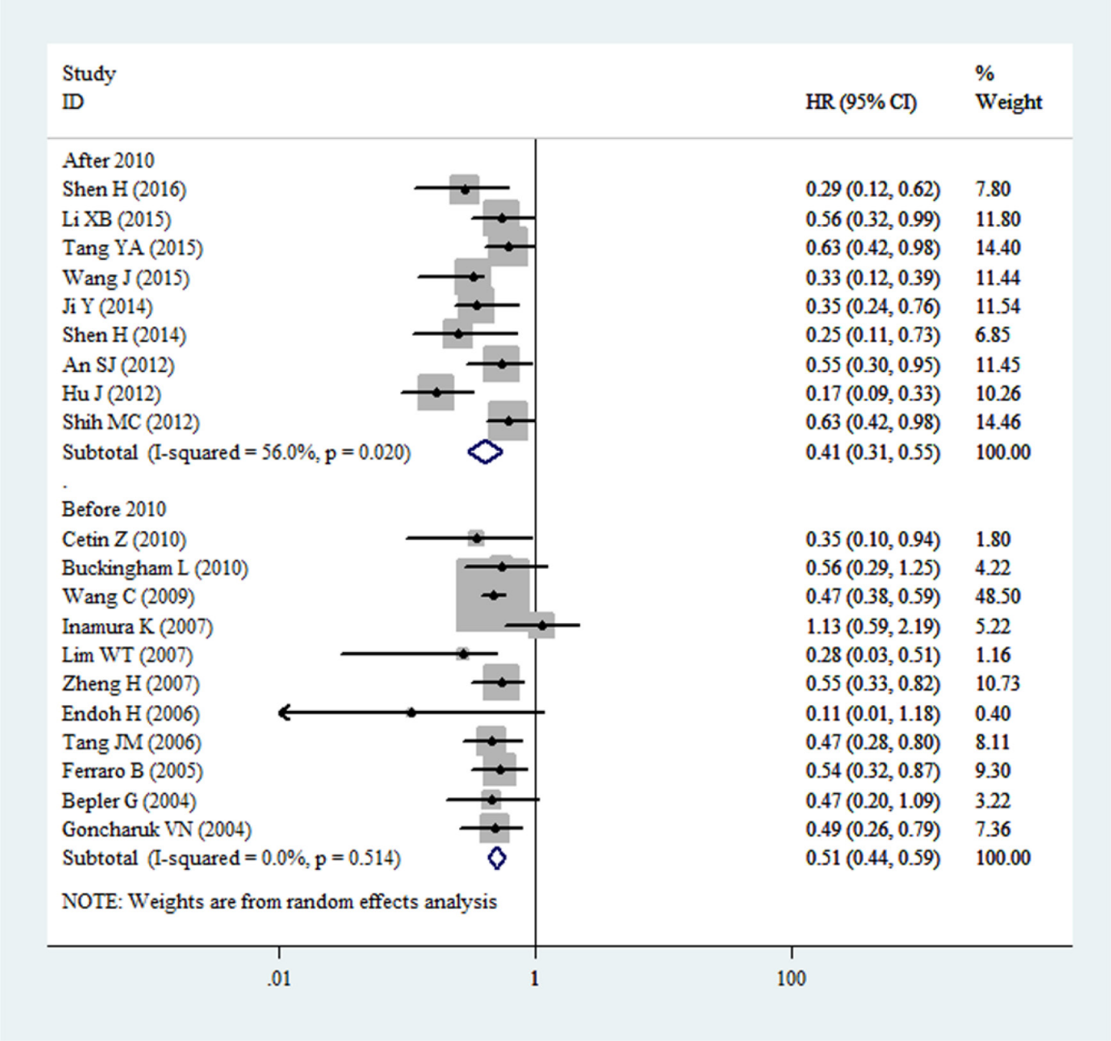

Supplementary Figure S5: Forest plot for the subgroup analysis of publication year. HR = hazard ratio; CI = confidence interval.

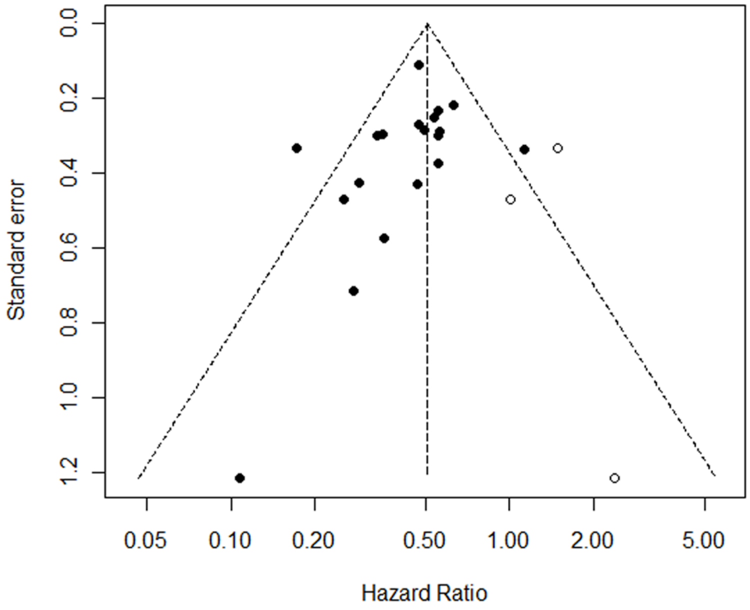

Supplementary Figure S6: Funnel plot for trim-and-fill method estimating the influence of potential publication bias.

Supplementary Table S1: Cut-off values of the decreased expression of PTEN

First author	Cut-off value
Shen H (2016) [13]	Negative or weakly positive expression
Wang J (2015) [14]	Negative (−) and weakly positive (+) expressions
Tang YA (2015) [15]	Immunostaining-positive tumor cells were less than 50%
Li XB (2015) [16]	The automated analysis system score of X-tile was less than 74.0
Ji Y (2014) [19]	Stained positive cells were fewer than 5%
Shen H (2014) [20]	The final IHC score was scored 0 or +
Hu J (2012) [21]	Less than 50% of the cells stained
Wang L (2012) [22]	Tissue specimens having a final score no more than 2
An SJ (2012) [23]	No staining intensity
Shih MC (2012) [24]	Immunohistochemistry scores of 0 and 1
Yanagawa N (2012) [25]	Complete absence of staining
Yoo SB (2011) [26]	The immunoreactivity staining was equal or less than 50%
Cetin Z (2010) [27]	Expression level in tumor tissue was less than normal tissue
Buckingham L (2010) [17]	The final scale was no more than 4
Wang C (2009) [28]	The percentage of reactive cancer cells was less than 5%
Inamura K (2007) [18]	Expression was less than 0.85-fold of the normal tissue
Zheng H (2007) [29]	The percentage of positive cells was 0–5%
Lim WT (2007) [30]	The sum of the intensity and extent scores was less than 2
Endoh H (2006) [31]	Low than the median expression
Tang JM (2006) [32]	Scoring produced 2 or less
Ferraro B (2005) [33]	Expression was less than the median value
Bepler G (2004) [34]	Expression was less than the median value
Goncharuk VN (2004) [35]	Low than moderate diffuse staining

Supplementary Table S2: Newcastle-Ottawa Scale (NOS) quality assessment of studies included in the meta-analysis

First author	Selection/4	Comparability/2	Outcome/3	Total score
Shen H (2016) [13]	3	0	2	5
Wang J (2015) [14]	3	2	2	7
Tang YA (2015) [15]	3	2	2	7
Li XB (2015) [16]	3	2	3	8
Ji Y (2014) [19]	3	2	3	8
Shen H (2014) [20]	3	0	2	5
Hu J (2012) [21]	3	2	2	7
Wang L (2012) [22]	3	2	2	7
An SJ (2012) [23]	3	0	2	5
Shih MC (2012) [24]	3	2	2	7
Yanagawa N (2012) [25]	3	2	2	7
Yoo SB (2011) [26]	3	1	2	6
Cetin Z (2010) [27]	3	1	2	6
Buckingham L (2010) [17]	3	0	2	5
Wang C (2009) [28]	3	0	2	5
Inamura K (2007) [18]	3	2	2	7
Zheng H (2007) [29]	3	1	2	6
Lim WT (2007) [30]	3	1	2	6
Endoh H (2006) [31]	3	0	2	5
Tang JM (2006) [32]	3	2	2	7
Ferraro B (2005) [33]	3	0	2	5
Bepler G (2004) [34]	3	2	2	7
Goncharuk VN (2004) [35]	3	0	2	5

Supplementary File S1: Search strategy

Updated until May 22, 2016.
Limited to original articles published in English.

Scopus: 57 results

TITLE("Phosphatase and tensin homolog" OR "Phosphatase and tensin homolog deleted on chromosome 10" OR "Phosphatase and tensin homolog deleted on chromosome ten" OR PTEN) AND (TITLE(NSCLC) OR (TITLE(lung) AND (TITLE-ABS(cancer OR tumor OR tumour OR neoplasm OR neoplasma OR neoplasia OR carcinoma OR adenocarcinoma OR cancers OR tumors OR tumours OR neoplasms OR neoplasmas OR neoplasias OR carcinomas OR adenocarcinomas)))) AND (TITLE-ABS(survival OR prognosis OR prognostic OR outcome OR survivals OR prognoses OR prognostics OR outcomes)).

Web of Science: 72 results

TI = (("Phosphatase and tensin homolog" OR "Phosphatase and tensin homolog deleted on chromosome 10" OR "Phosphatase and tensin homolog deleted on chromosome ten" OR PTEN) AND (NSCLC OR (lung AND (cancer OR tumor OR tumour OR neoplasm OR neoplasma OR neoplasia OR carcinoma OR adenocarcinoma OR cancers OR tumors OR tumours OR neoplasms OR neoplasmas OR neoplasias OR carcinomas OR adenocarcinomas)))) AND TS = (survival OR prognosis OR prognostic OR outcome OR survivals OR prognoses OR prognostics OR outcomes).

PubMed: 53 results

("Phosphatase and tensin homolog"[title] OR "Phosphatase and tensin homolog deleted on chromosome 10"[title] OR "Phosphatase and tensin homolog deleted on chromosome ten"[title] OR PTEN[title]) AND (NSCLC[title] OR (lung[title] AND (cancer[title/abstract] OR tumor[title/abstract] OR tumour[title/abstract] OR neoplasm[title/abstract] OR neoplasma[title/abstract] OR neoplasia[title/abstract] OR carcinoma[title/abstract] OR adenocarcinoma[title/abstract] OR cancers[title/abstract] OR tumors[title/abstract] OR tumours[title/abstract] OR neoplasms[title/abstract] OR neoplasmas[title/abstract] OR neoplasias[title/abstract] OR carcinomas[title/abstract] OR adenocarcinomas[title/abstract]))) AND (survival[title/abstract] OR prognosis[title/abstract] OR prognostic[title/abstract] OR outcome[title/abstract] OR survivals[title/abstract] OR prognoses[title/abstract] OR prognostics[title/abstract] OR outcomes[title/abstract]).

Embase: 55 results

("Phosphatase and tensin homolog":ti OR "Phosphatase and tensin homolog deleted on chromosome 10":ti OR "Phosphatase and tensin homolog deleted on chromosome ten":ti OR PTEN:ti) AND (NSCLC:ti OR (lung:ti AND (cancer:ab,ti OR tumor:ab,ti OR tumour:ab,ti OR neoplasm:ab,ti OR neoplasma:ab,ti OR neoplasia:ab,ti OR carcinoma:ab,ti OR adenocarcinoma:ab,ti OR cancers:ab,ti OR tumors:ab,ti OR tumours:ab,ti OR neoplasms:ab,ti OR neoplasmas:ab,ti OR neoplasias:ab,ti OR carcinomas:ab,ti OR adenocarcinomas:ab,ti))) AND (survival:ab,ti OR prognosis:ab,ti OR prognostic:ab,ti OR outcome:ab,ti OR survivals:ab,ti OR prognoses:ab,ti OR prognostics:ab,ti OR outcomes:ab,ti).

Supplementary File S2: Newcastle-Ottawa Scale (NOS) for quality assessment

Selection

(1) Representativeness of the exposed cohort
(a) Truly representative of the average patients with non-small cell lung cancer (NSCLC) in the community (1 star)
(b) Somewhat representative of the average patients with NSCLC in the community (1 star)
(c) Selected group of users (e.g., nurses, volunteers)
(d) No description of the derivation of the cohort
(2) Selection of the non-exposed cohort
(a) Drawn from the same community as the exposed cohort (1 star)
(b) Drawn from a different source
(c) No description of the derivation of the non-exposed cohort
(3) Ascertainment of exposure (proof of NSCLC and PTEN measurement)
(a) Secure record (e.g., surgical records or pathological diagnosis) (1 star)
(b) Structured interview (1 star)
(c) Written self-report
(d) No description
(4) Demonstration that outcome of interest was not present at start of study
(a) Yes (1 star)
(b) No

Comparability

(1) Comparability of cohorts on the basis of the design or analysis
(a) The age between exposed cohort and non-exposed cohort had no statistical differences (1 star)
(b) The sex (or grade, stage etc) between exposed cohort and non-exposed cohort had no statistical differences (1 star)

Outcome

(1) Assessment of outcome (death or recurrence)
(a) Independent blind assessment (1 star)
(b) Record linkage (1 star)
(c) Self-report
(d) No description
(2) Was follow-up long enough for outcomes to occur? (death or recurrence)
(a) Yes (24 months) (1 star)
(b) No
(3) Adequacy of follow-up of cohorts
(a) Complete follow-up: all subjects accounted for (1 star)
(b) Subjects lost to follow-up unlikely to introduce bias: small number lost (less than 25%) or description provided of those lost (1 star)
(c) Follow-up rate less than 75% and no description of those lost
(d) No statement

Note: a maximum of one "star" for each item within the "Selection" and "Outcome" categories, a maximum of two "stars" for "Comparability".
